# Achievements and Peculiarities in Studies of Ancient DNA and DNA from Complicated Forensic Specimens

**Published:** 2009-10

**Authors:** A.P. Grigorenko, S.A. Borinskaya, N.K. Yankovsky, E.I. Rogaev

**Affiliations:** 1 Vavliov Institute of General Genetics, Russian Academy of Sciences;; 2 Research Center of Mental Health, Russian Academy of Medical Sciences;; 3 University of Massachusetts Medical School, Worcester, U.S.A.

## Abstract

Studies of ancient DNA specimens started 25 years ago. At that time short mitochondrial DNA (mtDNA) fragments were the main targets in ancient DNA studies. The last three years were especially productive in the development of new methods of DNA purification and analysis. Complete mtDNA molecules and relatively large fragments of nuclear DNA are the targets of ancient DNA studies today. Ancient DNA studies allowed us to study organisms that went extinct more than ten thousand years ago, to reconstruct their phenotypic traits and evolution. Ancient DNA analyses can help understand the development of ancient human populations and how they migrated. A new evolutionary hypothesis and reconstruction of the biota history have been re-created from recent ancient DNA data. Some peculiarities and problems specific to the study of ancient DNA were revealed, such as very limited amounts of DNA available for study, the short length of the DNA fragments, breaks and chemical modifications in DNA molecules that result in "postmortem" mutations or complete blockage of DNA replication in vitro. The same specific features of DNA analysis were revealed for specimens from complicated forensic cases that result in the lack of experimental data or interpretation problems.. Here, we list the specific features of ancient DNA methodology and describe some achievements in fundamental and applied research of ancient DNA, including our own work in the field.

## INTRODUCTION

Ancient DNA studies allow to empirically verify evolutionary hypotheses and contribute to the complex reconstruction of historical changes in biota. The analysis of DNA from human archeological samples reveals information on the genetic traits of ancient inhabitants of various geographical regions.

**Fig. 1.**
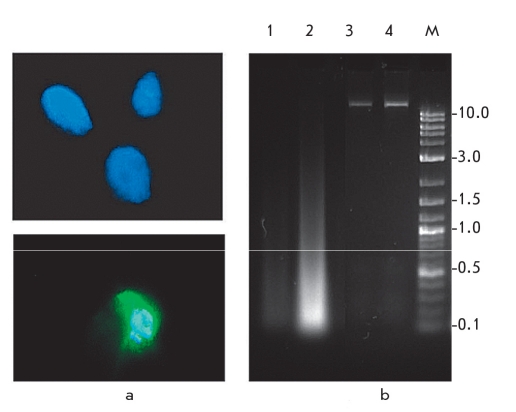
Unusually well-preserved mammoth M. primigenius DNA specimen obtained from permafrost-preserved remains found in 1986 in the valley of the Enmynveem River (Chukotka): a - Fluorescence of nuclei in muscle cells of M. primigenius (~33, 000 years old) after DAPI staining indicates a relatively high degree of DNA preservation; b - electrophoresis of total genomic DNA extracted from mammoth muscle tissue (lane 1 is 1/10 dilution of the DNA on lane 2), and control DNA isolated from fresh human blood samples (lanes 3 and 4). The right lane is a DNA molecular weight marker (fragments sizes are indicated in kb) [13]

The first published reports on the study of ancient DNA appeared 25 years ago. Researchers managed to extract a DNA fragment from a museum sample of dried muscle tissue taken from a quagga - a South-African odd-toed ungulate animal that disappeared in the 19^th^ century. The extracted DNA fragment was cloned in a phage vector and sequenced. Phylogenetic analysis showed that the determined sequence of the mitochondrial DNA (mtDNA) was related to zebra species [1, 2]. The next study described the extraction, cloning, and sequencing of DNA fragment from a ~2,400-thousand-year-old Egyptian mummy [3]. After these, attempts were made to extract DNA from the remains of animals, plants and microorganisms whose ages ranged from several hundreds to more than a million years (see review in [4]). As the data accumulated, it became clear that the age of the remains that could still have analyzable templates, calculated using kinetics of DNA decay, was not greater than 0.1-1.0 Myr, and that the level of DNA preservation depended on the age and type of the biological sample, and also on the conditions in which it was preserved [5, 6]. Reports on the extraction of DNA from specimens older than 1 Myr are most probably erroneous. The most ancient authentic DNA samples have been isolated from permafrost specimens, such as mammoth, bison and other animal remains, chloroplast DNA from plants, and bacterial DNA [7-12]. Fragments up to 900-1 000 base pairs (bp) have been amplified from these samples. The low temperature and humidity improve DNA preservation, that allows researchers to analyze samples from remains that are tens of thousands of years old (Fig. 1) [13].

Studies of DNA isolated from ancient or historic specimens must deal with a number of methodical problems. These include the exceedingly small quantity and small size of the DNA fragments extracted from ancient samples, as well as the presence of chemical DNA modifications that block DNA replication and cause the appearance of postmortem mutations in the nucleotide sequence. Spontaneous damage of DNA molecules in a living cell is repaired during replication or causes death and elimination. After an organism dies, both the reparation and elimination of cells with damaged DNA come to a halt; that leads to the accumulation of chemical modifications and fragmentation of DNA molecules. Furthermore, the DNA is further destroyed by the organisms of the soil biota. Degradation of ancient DNA makes contamination of the samples by even a single molecule of modern DNA a major cause of false results.

Using the PCR techniques [14-16] has considerably widened the possibilities of ancient DNA analysis, since it allows in vitro amplification of a single original DNA molecule. PCR allows selective amplification of target DNA fragments; that is very important in ancient DNA analysis, since 99% of the extracted DNA can be a mixture of bacterial or fungal DNA from soil.

The development of extraction and sequencing technologies in ancient DNA study in the past few years has allowed to sequence the complete mitochondrial genomes, to reconstruct nuclear genomic regions, to analyze the genetic population variety of extinct species (moa, mammoth, furry rhinoceros, cave bear, Beringian bison, giant eagle, Neanderthal, etc.), and to study changes in the Pleistocene and Holocene ecosystems (see reviews [4, 17-20]).

Years of research have resulted in the establishment of a number of requirements to ancient DNA studies and criteria upon which the authenticity of resulting data is judged. Contamination of the analyzed samples by modern DNA is still one of the most pressing problems during ancient DNA analysis. One of the well-known examples is the report related to the extraction of a DNA fragment from the dinosaur bone [21] that, as it turned out during further analysis, was a fragment of human nuclear DNA [22], as well as the mentioned above attempt to sequence DNA from an Egyptian mummy [3]. Currently, the sequence obtained in the mummy study is assumed to be the result of modern human DNA contamination [17, 23].

DNA sequences reconstructed from ancient or historic samples can also contain errors because of hydrolytic or oxidative modification of the ancient DNA. For example, sequencing of long regions of the same sample of Neanderthal nuclear DNA was performed by two groups of researchers. The Edward Rubin group from the Joint Genome Institute of the U.S. Department of Energy published a 65,000 bp sequence, and the Svante Paabo group from the Max Planck Institute for Evolutionary Anthropology reported the sequencing of 1 million bp [24, 25]. Further data analysis determined that a considerable amount of errors was present in the second group's data. Most of the data was the result of contamination by modern DNA. Furthermore, the "single-run" sequencing approach used by this group did not prevent multiple errors that appeared because of ancient DNA nucleotide modification and could only be excluded by multiple sequencing [26-28]. Errors were also detected in the published sequence of the mtDNA fragment from the Feldhofer cave Neanderthal [29]. Out of the 27 detected alterations, as compared to human mtDNA, 4 turned out to be artefacts [30]. The published nucleotide sequences of other species are also not free of errors (the Pleistocene cave bear [31], mammoth [32, 33], etc.).

Similar problems (exceedingly small amounts of DNA, or DNA damaged by thermal conditions or chemical agents) often come up during the genetic analysis of forensic samples. An analysis of these problems and approaches that allow to solve them are presented in this review.

## EXTRACTION OF DNA AND CONTAMINATION PROBLEM

Paleontological and archeological materials and biological samples that are collected at excavation sites or stored in museums yield very small amounts of DNA that is usually highly fragmented. Moreover, this ancient DNA is modified in various ways that prevents amplification or lead to errors in nucleotide sequence reads. Because of the low efficiency of amplification of authentic DNA extracted from ancient and historic samples, contamination of the sample by even a single modern DNA molecule can produce errors. A number of specific measures must be taken in order to prevent contamination and to detect possible contamination. False positive results, caused by in-lab contamination, are one of the major problems in ancient DNA studies. That is why the key step in molecular-genetic analysis of ancient and historic samples is DNA extraction.

Extraction of DNA from ancient samples must be performed in accordance with sample age and quality. It particularly involves the choice of the detergent used for cell lysis. Sodium dodecyl sulfate (SDS) that is used in standard DNA extraction for the purpose of lipid destruction could be substituted by non-ionic detergents for soft lysis (Triton or Twin), or by detergent-free extraction, since lipids have been already destroyed in ancient samples, and the use of SDS lowers the DNA yield. Nevertheless, the use of detergents is recommended for more recent samples. Treating bone material with reagents that include EDTA causes sample decalcification and pH lowering, which can affect DNA binding on the columns used in downstream extraction procedures.

Studies of ancient DNA should be conducted in specially equipped facilities with all possible means for preventing contamination by modern DNA. It includes facilities with altered air pressure: high pressure in the rooms used for ancient DNA work and low pressure in rooms where modern DNA and amplified products are studied. These facilities must be regularly disinfected with chemicals and UV-radiation, to avoid any DNA (target, amplified or contaminant) and cell material (aerosols and dust with microorganisms and human and other organism's cells). Ancient DNA work must be conducted in protective clothing, gloves, and masks. A minimum requirement is that DNA extraction procedures performed on ancient DNA and involving single fragmented molecules must be performed in facilities physically separated from the ones for PCR-amplification and downstream amplified DNA procedures that work with millions of molecules. Facilities for ancient DNA handling must not house procedures with amplified fragments, as it is exceedingly difficult to prevent their spreading. DNA from modern organisms must be handled in a separate building, or at least a facility with a separate ventilation system. All these measures help to prevent contamination but do not affect the contamination of the sample itself that remains as it was before it ever got into the laboratory. In order to decrease contamination, the surface layer of the sample is usually removed.

Contamination is usually a very important issue in ancient human or microbal DNA studies, since both human and bacterial DNA are constantly present in laboratory. Thus, the sequences of contaminant DNA are harder to separate from the authentic DNA than when dealing with exotic or rare species. The guidelines for working with ancient DNA and the criteria of authenticity for the amplified ancient DNA fragments are described in the following reviews [4, 17, 34-36] and are listed in Table 1.

**Table 1:** Authenticity criteria for ancient DNA

Criteria	Importance for authenticity
Reagents and plastic to be used for work with ancient DNA must be checked for the possible presence of amplifiable templates. Since the templates can be present in trace amounts, and thus be amplified in only one out of several samples, multiple checks have to be performed.	Prevents contamination through reagents and disposable materials.
All the manipulations that are used to extract DNA are performed on solutions with no templates using the same solutions. PCR is performed with a double negative control; the normal one (reaction mix with no template) and the reaction mix with the "empty" extract.	Helps detect contamination which could have happened during extraction or during the PCR mix preparation.
Positive controls are usually not used, since they carry the risk of potential contamination.	Prevents contamination.
When possible, several independent extractions of DNA are performed from different areas of the sample.	Helps identify local contamination of the sample itself.
Repeated amplifications of material obtained from the same and from different extractions.	Helps identify sporadic contamination and facilitates the identification of erroneous nucleotides which were included into products amplified from degraded DNA extracts with a small amount of template molecules.
Cloning of amplification products and/or sequencing of multiple clones.	Identifies heterogeneity in the amplified products, which stems from contamination or amplification of degraded DNA with modified nucleotides.
Determination of the number of amplified DNA template molecules (must be determined for each pair of primers, since the number of amplified molecules can vary noticeably depending on the length and the nucleotide content of the amplified fragment, and also on the sensitivity of the specific pair of primers).	Determines the possibility of insertions of nucleotides not present in the original sequence. Extracts which contain only a few or even a single molecule are very much prone to yield erroneous insertions, so it is necessary to perform several amplifications. Extracts which contain at least 1,000 molecules require only one amplification reaction.
Peculiar "molecular behavior," a reverse correlation between the efficiency of amplification and the length of the amplified fragment .	If the sample does not exhibit more intensive amplification of shorter fragments than that of longer fragments, as compared to modern DNA, this indicates that the source of the amplified DNA is contaminated by modern templates.
Biochemical analysis of the level of preservation of macromolecules.	A high level of biochemical preservation of macromolecules indicates a high probability of preserved DNA molecules being found in the sample. This DNA can be analyzed. Thus, the test would support the authenticity of the sequencing results.
Avoid introducing nuclear sequences into mtDNA.	Nuclear DNA has regions homologous to mtDNA, so this fact must be taken into account if mtDNA amplification is used.
Independent confirmation of results in a different laboratory.	This helps identify laboratory contamination of samples or reagents, but it does not rule out contamination that was present in the sample itself (contaminants which were a part of the sample before it arrived at the laboratory, for instance during archeological excavation). This requirement used to be mandatory. Now it has been dropped.

Identifying contamination

Possible contamination can be identified with a high degree of accuracy during ancient DNA studies if it is a priori supposed that the sample could be contaminated, so that the results are viewed with this possibility in mind. In order to identify laboratory contamination, researchers use "empty" extracts that have been processed along with the target sample but without adding tissue sample or DNA. Since contamination templates can be present in very low concentrations and not manifest themselves in each reaction, multiple control reactions are made, usually in proportion 1:5 , but with no fewer than 1:1 to the extracted sample. Such "empty" extracts are used in all further analytic procedures in addition to the regular negative controls.

Independent confirmation of results in different laboratories is considered to be one of the strong indicators of authenticity. But even this is not an absolute guarantee [36].

Special attention must be paid to bioinformatic analysis of the obtained nucleotide sequences. Since analysis of ancient samples usually involves mtDNA analysis, it is important to compare the sequence not only with the mtDNA of species closely related to the sample source or with human mtDNA (the most likely source of contamination), but also with the nuclear homologues of mtDNA genes (nuclear mtDNAs, numts), whose similarity to mtDNA is ~98% and more in case of human mtDNA (such as the NT_004350.18 sequence located on Chromosome 1).

## CHEMICAL MODIFICATIONS OF ANCIENT DNA AND POSTMORTEM MUTATIONS

**Fig. 2.**
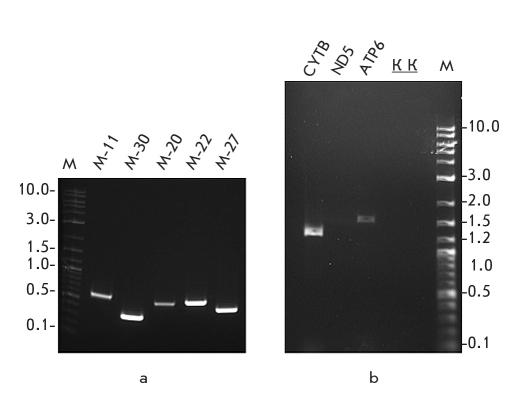
A typical result of PCR amplification of mammoth mitochondrial genome fragments: a - PCR products of relatively short amplification fragments (300-600 bp); b - Successful amplification of long PCR fragments harboring entire mitochondrial gene sequences (1317 bp for the CytB gene and 1613 bp for the ATP6 gene) and no amplification products are obtained for larger size PCR fragments (3054 bp for the ND5 gene). M - marker fragment sizes are indicated in kb, K - negative controls [13]

Postmortem DNA alterations, and mutations during in vitro DNA amplification, are among the central methodological problems in ancient DNA and complex forensic DNA analysis. As opposed to metabolically active tissues that have an active DNA reparation system postmortem cells accumulate chemical (hydrolytic or oxidative) DNA modifications and strand damage. Studies show that postmortem DNA damage includes strand breaks, loss of bases and cross-linking between strands that inhibits PCR. Postmortem alterations that modify bases but do not inhibit amplification are especially important, since they can cause the appearance in the amplification products nucleotide of changes that were not present in the authentic sequence (type I substitutions A > G / T > C and type II substitutions C > T / G > A) (Table 2). The manner how the degraded DNA templates are damaged depends on the samples age, their geographic origin, and the taphonomic conditions (preservation conditions) of the environment where the samples were stored. Postmortem alterations can appear in mutational hot-spots, thus simulating evolutionary mechanisms [37]. The manner and dynamics of accumulation of postmortem DNA alterations are under continuous research [38, 39]. DNA damage limits the size of the DNA fragments found in ancient samples to about 100-500 bp. That is why the primers for ancient DNA PCR are usually chosen for no more than 200-300 bp fragments, although fragments of greater length have been obtained in some cases (Fig. 2).

**Table 2:** Various types of ancient DNA damage (from [4, 17] with modifications).

Type of damage	Cause of damage	Effect on DNA	Possible solution
Nucleobases and deoxyribose degradation	Postmortem destruction by intracellular nucleases, degradation by microorganisms and other chemical processes	Apurinization of DNA, strand breaks, decrease of DNA fragment size, decrease of the overall amount of DNA	Amplification of short (<100-200 bp) overlapping fragments
Cross-links which block PCR	Alkylation, Maillard reaction (chemical reaction between a sugar molecule and an amino group of a nucleobase or an amino acid)	Cross-links between DNA strands in a single molecule; cross-links between DNA strands of different molecules; or cross-links between DNA and proteins	Treating the sample with reagents that destroy cross-links
Deamination and other types of oxidative or hydrolytic DNA base modifications	Adenine → hypoxanthine Guanine → xanthine Cytosine → uracil 5-methyl-cytosine → thymine	Insertion during amplification of nucleotides that were not present in the original nonmodified template	Treatment by DNA uracil-N-glycosylase, which removes cytosine deamination products. Determination of a consensus sequence based on multiple sequencing of the analyzed regions: Multiple independent PCR, cloning of the original template or PCR products, and sequencing of several clones

**Fig. 3.**
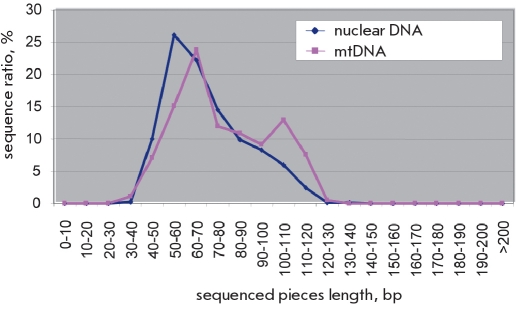
The size distribution of genomic DNA fragments extracted from the ancient remains sequenced on the 454 platform. Unpublished data obtained in collaboration of E.I. Rogaev et al. with M. Blow and E. Rubin

Most of the published ancient DNA studies have been conducted on mtDNA that is found in hundreds and thousands of copies in cell and can be amplified more successfully than nuclear DNA. There are much fewer studies on nuclear DNA. Amplification, cloning, and sequencing of nuclear DNA from a mammoth M. primigenius sample obtained from Chukotka permafrost was performed in order to assess the quality of nuclear DNA preservation (E.I. Rogaev, E. Rubin, unpublished data). Most of the mammoth genome was fragmented into pieces of about 50-100 bp (Fig. 3). It indicates a relatively high quality of nuclear DNA preservation.

**Fig. 4.**
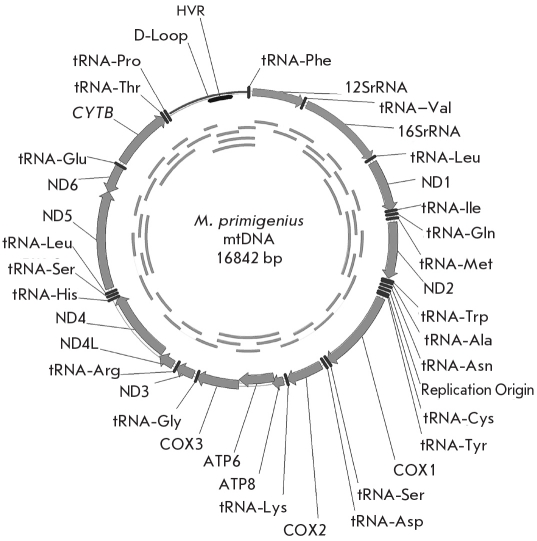
Mitochondrial genome of the woolly mammoth M. primigenius. Determination of the nucleotide sequence of the complete mitochondrial genome was performed in two laboratories. The overlapping PCR-amplification products used for sequencing are shown in the inner circle [13]

Postmortem modifications are randomly located in the preserved DNA fragments. For example, single nucleotide substitutions (~ 6 in 1 000 bp) were found in one study [13] during the cloning and sequencing of PCR-amplified mammoth DNA. This observation was taken into account for correct reconstruction of the complete mitochondrial genome of Chukotka mammoth M. primigenius (Fig. 4). The complete genome was obtained as a consensus of multiple overlapping fragments [13]. In order to additionally control the number of postmortem mutations, the overall number of substitutions in all mammoth mtDNA genes was calculated in comparison with elephant E. maximus mtDNA. The ratio between nonsynonymous (that cause aminoacid substitutions) and synonymous substitutions was calculated for the same purpose. It was shown that the number of substitutions in the mtDNA genes of the Chukotka mammoth [13] was lower than in the genes of a mammoth sequence obtained from remains found in Yakutia that was published at the same time by German researchers [40]. A comparative analysis showed that this difference was due to the unusually high number of substitutions in a 200-300 nucleotide region of the Yakut mammoth DNA in the locus of ND1 and ND2 genes, and that the number of nonsynonymous substitutions was greater than the number of synonymous ones (2:1 for the ND1 gene and 7:2 for ND2). The ND2 gene of the Chukotka mammoth had only one synonymous substitution, and ND1 did not differ in any way from the elephant gene [13]. All undetected postmortem mutations affect the result of phylogenetic reconstruction.

## NOVEL DNA-SEQUENCING TECHNOLOGIES

Ancient DNA analysis involves sequencing of a large number of short fragments that have multiple overlapping of the same genomic regions. Low sequencing speed and high cost limit the usage of such research. Novel technologies of massively parallel sequencing of a large amount of DNA samples have appeared in the last 3-4 years, and the cost has dropped by two orders of magnitude. This novel technologies have given researchers sequencing possibilities that were previously available only to large genomic centers. Among the available novel technologies several were used in ancient DNA studies, such as clonal amplification followed by parallel sequencing of dense micropanels of cloned DNA fragments by repeated enzymatic reaction cycles, with automatic registration of the signal from each cycle and every fragment.

The ordered spatial localization of the PCR amplicons on a chip or immobilization on microne-sized beads that are then placed into ordered cells allows to minimize the reaction mix volumes, thus decreasing the cost of the whole process.

Realization of these strategies involves several steps, and for each of them a specific technical approach has been developed. For example, preparation of DNA fragment libraries using PCR does not keep the ratio of amplification products identical to the ratio of original templates. Some DNA fragments are amplified more efficiently than others that could be lost. The problem can be solved by using emulsion PCR. The DNA solution is transferred into the mineral oil mix. The ratio is calculated so that each molecule of DNA is encapsulated in a separate lipid droplet that acts as a microreactor for the amplification process. This approach minimizes the loss of certain original templates. There are various technical solutions for fragment library preparation and for the other steps in the process, such as enzymatic reactions, visualization and computerized signal registration, data storage and analysis [41].

Novel sequencing technologies have certain limitations. Massively parallel pyrosequencing, accomplished by using 454 Life Science system (Genome Sequence 20TM DNA sequencing System: GS20, Roche/454 Life Science), provides a 100-fold increase in the sequencing speed, compared to the standard capillary electrophoresis method. Up to 25 million nucleotides are analyzed in a single run. Only a short sequence can be read (usually less than 250-400 bp). It is not much of a limitation for ancient DNA studies, since most of the DNA fragments are precisely of this size..

The Illumina technology, named Solexa (after the name of the company that developed this approach), and SOLiD (ABI corporation) allow the analysis of up to 1 billion nucleotides in a single run but read sequences of only 30-40 nucleotides (last year this number was just 25 nucleotides). The availability of full human genomes and the genomes of commonly used model organisms as reference sequences allow to map the short fragments into a single contig.

Another limitation of these novel platforms is the 10-fold decrease in the accuracy of sequencing, compared to the Sanger method. Nevertheless, these technologies are very promising, and they can be expected to improve in efficiency and quality in the nearest future.

## ANALYSIS OF DEGRADED DNA IN FORENSIC EXPERTISE OF HISTORIC SAMPLES

The technological approaches applied for ancient DNA study can also be used for forensic genetic analysis in difficult cases where only micro-scopic amounts of material are available or the DNA has been severely damaged. Some of these approaches were used in the genetic expertise of the putative remains of the family of the last Russian Emperor Nicholas II Romanov. In the early 1990s, a first grave with human remains was found near Yekaterinburg. During the investigation, it was suggested that the remains belong to the family of the Russian Emperor Nicholas II Romanov, his wife, the Empress Alexandra Fedorovna, their 3 daughters, the court physician, and three servants. They are all thought to have been murdered in 1918 [42-44]. They are all thought to have been murdered in 1918 [42-44]. However, the remains of two children of the Romanov family were not identified, and their fate remained unknown. Among other hypotheses, there has been a legend that Alexey and Anastasia, the youngest children of the Romanov family, had survived those turbulent times. In July 2007, a second grave was found not far from the first one. It contained burned bone fragments from two skeletons. Forty-four bone fragments were found in the second grave, all severely damaged by fire and presumably sulfuric acid. Preliminary anthropological analysis of the half-burned bone fragments from the second grave suggested that the bones belonged to a boy 10-14 years of age and a young woman of about 18-23. The least damaged fragments of the femoral bones from both the male and female skeletons were selected for genetic analysis, and they were labeled Samples 146 and 147, respectively. Samples from the first grave were also collected for a more detailed study, and reference samples were taken from living relatives of Nicholas Romanov and Alexandra Fedorovna. Furthermore, swabs of blood stains from a shirt that had belonged to Nicholas II and is stored in the Hermitage museum were also used for analysis. The study included the following steps: preparation of the samples for DNA extraction; DNA extraction; quantification of the extracted total DNA and human-specific DNA; amplification and sequencing of the mitochondrial hyper-variable regions, and later sequencing and reconstruction of the complete mtDNA (cmtDNA) sequence; determination of the STR-profiles of the Y-chromosome; determination of the autosomal STR-profiles; additional sex identification with the use of a special marker designed for degraded DNA analysis [45, 46]; and extraction and analysis of modern DNA from Romanov family relatives and their comparison to historic samples. The steps and methods of DNA identification are described in Table 3 [45].

**Table 3:** Methodical approaches specific to analyses of degraded DNA from historical specimens [45]

Stage of analysis	Special procedures	Reagents and methods
Preparation of historic samples	Independent analysis in specialized laboratories in IOGene (Moscow) and University of Massachusetts Medical School (Worchester, USA).	Physical and chemical cleaning of small bone fragments, crushing or drilling to obtain bone powder.
Extraction of DNA from bone remains	All the experimental procedures were performed in sterile PCR-hoods, in accordance with standards for ancient DNA research, keeping to all the safety precautions so as not to contaminate the samples by modern DNA.	DNA was extracted from ~170≤700 mg of cleaned bone material treated by 0.5 M EDTA and proteinase K and was then purified by a QIAquick PCR purification kit (Qiagen) in accordance with the manufacturer's protocol with slight modifications.
Extraction of DNA from archive spots of blood	The biological material was obtained from 4 different blood stains. At least 3 swabs were taken from each spot. In order to minimize contamination, DNA was extracted only from the 2nd and 3rd swabs of each spot.	DNA was extracted with the QIAamp DNA Mini Kit (Qiagen) in accordance with the manufacturer's protocol ("DNA Purification from Dried Blood Spots") with several modifications.
Quantative DNA analysis		The total DNA was quantified by the Quant-iT™ PicoGreen® Assay kit (Invitrogen), human specific DNA was quantified by the Plexor® HY assay kit (Promega) and the 7500 Real-Time PCR System (Applied Biosystems).
Sequencing HVR1 and HVR2 of mtDNA from historic samples	Possible contamination by foreign DNA was monitored by using negative controls (amplification of "empty" extracts and PCR without addition of the template).	mtDNA fragments were amplified as short overlapping fragments. The PCR products were then extracted from the agarose gel using a QIAquick Gel Extraction kit or a MinElute Gel Extraction kit. For additional studies, the PCR products of samples from the second burial site were cloned.
Sequence analysis of the complete mitochondrial genome, extracted from bone remains.	Since the DNA was highly degraded, short overlapping fragments sized 164-383 b.p. were obtained, covering the whole mitochondrial genome.	Because the amount of DNA was initially so small, multiplex amplification was performed using 88 pairs of specially developed primers grouped into 3 kits, and then the products of this PCR were amplified with individual primer pairs. The PCR products were then sequenced using three different strategies
Analysis of the mtDNA extracted from the blood stains on Nicholas the Second's shirt.	Up to 5 or 7 repeated PCR reactions were conducted for some of the SNPs.	Since the quality of preservation in the blood stains was unknown, a kit of primers was developed for the amplification of short (64≤109 b.p.) DNA fragments, which would include very rare SNPs identified in the previous analysis of Skeleton №4 (the putative skeleton of Nicholas the Second).
Extraction and analysis of DNA from modern samples.	All the procedures involved in analyzing modern DNA were performed in other buildings, which were located some distance away from the ancient DNA laboratories. All the living relatives who took part in the study gave their written consent.	DNA obtained from buccal swabs or drops of blood was extracted using standard protocols. PCR was performed using a kit of primers for amplifying longer fragments.
Assembly of fragments into a continuous sequence of nucleotides (contigs).		The sequences were assembled using Seqman software, DNASTAR, and the revised Cambridge reference sequence (rCRS, accession number AC_000021) as a standard.
Sex identification.		Sex was identified using the standard method, amplification of a fragment of the amelogenin gene using several commercial kits: AmpF≤STR® MiniFiler™ (Applied Biosystems) and PowerPlex S5 (Promega). Specially developed primers for the amplification of short fragments specific to the X- and Y-chromosomes were also used.
Analysis of nuclear STR markers.	During the initial study, mtDNA or nuclear DNA extracts that consisted of a mix of individual profiles were discarded from further analysis. Each sample from various extracts was serially amplified. Homozygous loci were considered authentic if multiple independent amplifications confirmed a certain allele for the autosome STR-marker.	The following kits were used in order to obtain autosomal STR profiles from bone samples of the first and second burial sites: AmpF≤STR® MiniFiler™ PCR Amplification Kit (Applied Biosystems) and PowerPlex S5 System (Promega), developed especially for analyzing degraded DNA.
STR-profiles of the Y chromosome.		The AmpF≤STR® Yfiler™ (Applied Biosystems) kit was used, according to the manufacturer's protocol with slight modifications for work with degraded DNA.
Electrophoresis analysis	In order to increase the signal intensity and lower "noise" in the STR-profiles, the products of multiplex amplification were sometimes purified according to the method for genotyping low-copy DNA templates using Qiagen MiniElute PCR purification kit.	Electrophoretic analysis was performed with a 96-capillary sequencer 3730xl DNA Analyzer (Applied Biosystems). The results were analyzed using GeneMapper ® ID software v3.2 (Applied Biosystems).

## MITOCHONDRIAL GENOME ANALYSIS

Complete nucleotide sequences of the mitochondrial genome have been determined for the putative remains of Nicholas II and Alexandra Fedorovna from the first grave; and the putative remains of Alexey and his sister, from the second grave. The mitotypes of the putative remains of Nicholas II and Alexandra Fedorovna are from the common European mtDNA haplogroups T2 and H1.

**Fig. 5.**
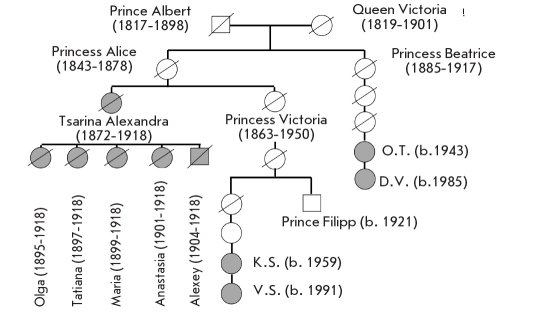
Maternal lineages of Empress Alexandra Fedorovna. The family members whose mitochondrial DNA was determined are indicated in grey

Complete cmtDNA sequences were also determined for the relatives of Queen Victoria (1819-1901) for 2 maternal lineages, the descendants of princess Victoria, sister of Alexandra Fedorovna, and their aunt Beatrice (Fig. 5). Their cmtDNA were absolutely identical with those extracted from the putative remains of Alexandra Fedorovna and the 2 children from the second grave. Searches performed in the available cmtDNA databases (Table 4) showed that not one of the available tens of thousands of sequences identifies with this cmtDNA, which was named "Queen Victoria mitotype." Thus, the first and second burial sites really do contain the remains of Queen Victoria's granddaughter, great granddaughter, and great grandson.

**Table 4:** Databases used for analyzing the population frequencies of the determined genetic profiles [45-49]

Query sequence	Number of samples in the database	Name of the database
Complete mitochondrial genome	71,664 2,704 14,486	Mitosearch (www.mitosearch.org) contains sequences of the hypervariable region; mtDB, Human Mitochondrial Genome Database (www.genpat.uu.se/mtDB); EUROS, our own database, compiled from our own and other authors' published data for the USSR and Russian populations and for certain European populations (German, English, etc.).
STR-haplotype of the Y-chromosome	4,163 1,261	U.S.Consolidated Y-STR Database, which has genotypes for 15-17 STR loci (http://www.usystrdatabase.org/); Data for Russians from the Y Chromosome Haplotype Reference Database (YHRD, www.yhrd.org/index.html).
Autosome STRs	> 50,000	For this study we combined the nonoverlapping data for European populations from two large databases: ALFRED (http://alfred.med.yale.edu/alfred/) and The Distribution of the Human DNA-PCR Polymorphisms database (http://www.uniduesseldorf.de/WWW/MedFak/Serology/database.html) and also added the recently published data on the Russian population.
SNP in the IVS3-3 position of the F9 gene		Population SNP database (NCBI, HapMap Project); ~ 928 X-chromosomes of healthy individuals of European descent whose genotypes were assessed for this SNP using PCR-RFLP (Restriction Fragment Length Polymorphism) especially for this study; Hemobase: Hemophilia B mutation registry, Haemophilia B mutation database.

Determination of the cmtDNA from the putative remains of Nicholas II confirmed the earlier described heteroplasmy (the coexistence of several mtDNA types) at the 16169C/T site. Moreover, nucleotide substitutions were found in the coding region of the mtDNA, including the extremely rare 2850C variant in the 16S rRNA gene (population frequency approximately 0.004). Nucleotide substitutions in the mtDNA from the putative remains of Nicholas II and from the blood stains on the shirt were completely identical [45]. The ratio between the heteroplasmic mtDNA variants was similar both in the remains and in the blood. Nicholas II's brother, George, whose remains were studied previously, also had heteroplasmy at the same site [43]. The descendants of Kseniya, sister of Nicholas II, had the homoplasmic 16169 T variant, and the previously performed mtDNA study of Nicholas II's sister's son found the homoplasmic 16169C variant [44]. Not one of the reconstructed complete mtDNA sequences ("Dagmar" type) was identified in available databases (Table 4), which confirms the assumption that the studied remains belong to Nicholas II. The data suggest that the brothers Nicholas II and George Romanov inherited heteroplasmy from their mother Maria Fedorovna (Princess Dagmar), and that her descendants had this heteroplasmy segregated into two distinct mtDNA variants in two generations. The nucleotide substitution at position 16169, on which the heteroplasmy of the "Dagmar type" is based, is located in the noncoding (hypervariable) region of the mtDNA characterized by increased polymorphism compared to the coding regions. The population frequency of heteroplasmy for point substitutions in hypervariable regions averages 6% [47]. It is assumed that rapid segregation of heteroplasmic mtDNA variants in descendants is caused by mechanisms of biological "bottle-neck" (a massive decrease in the number of mtDNA copies) during oocyte maturation in the postnatal folliculogenesis in mammals [48].

## NUCLEAR STR-MARKER ANALYSIS

**Fig. 6.**
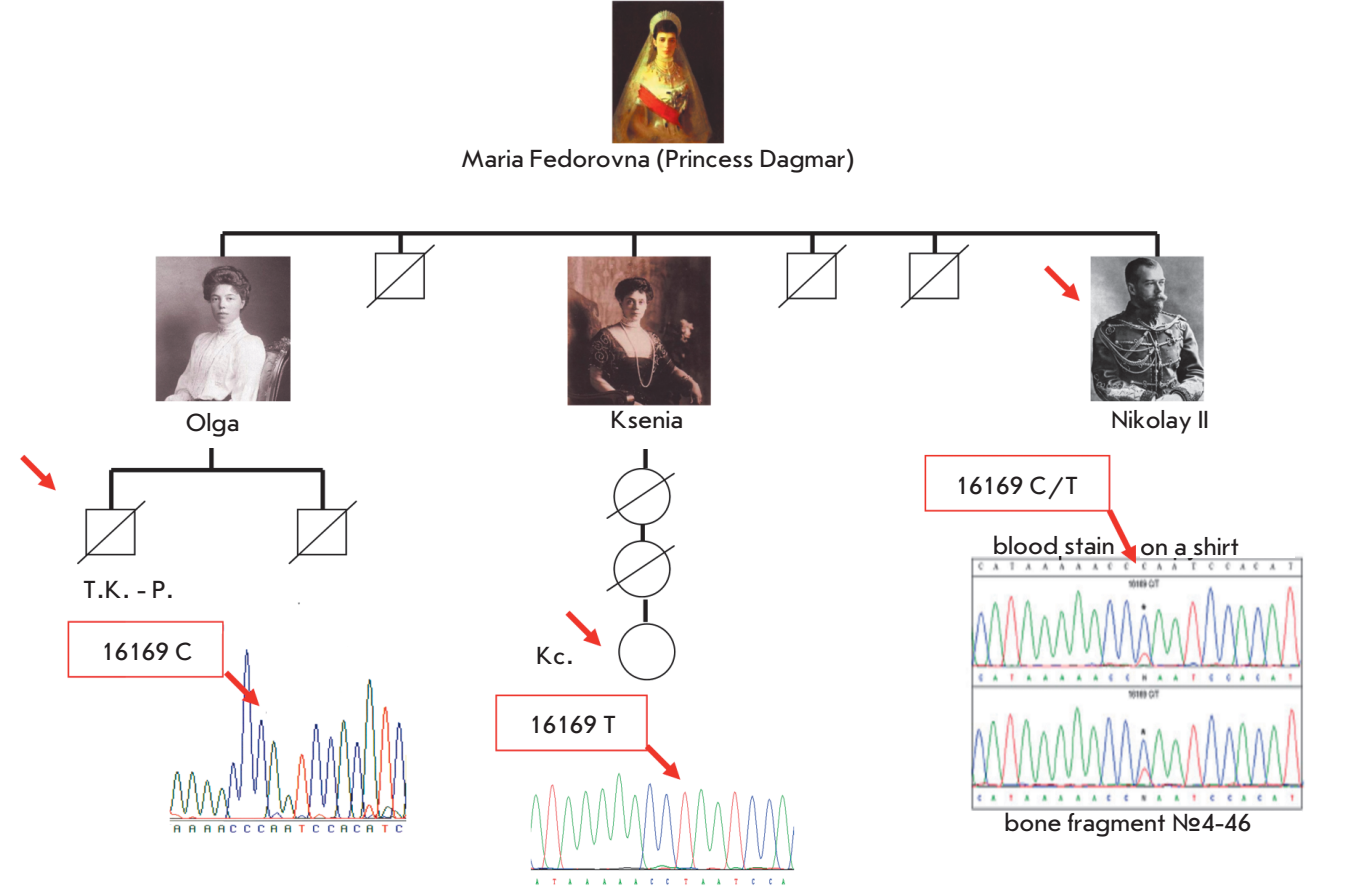
Heteroplasmy at the 16169C/T locus in the mitochondrial (maternal) lineages of Emperor Nicholas II

**Fig. 7.**
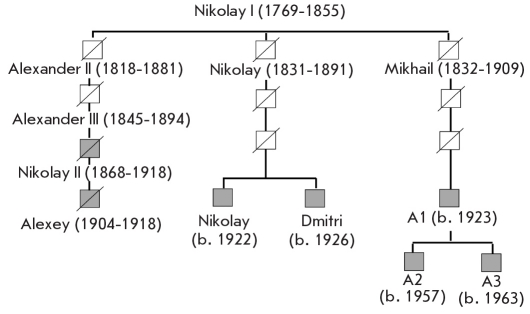
The paternal lineages of the Romanov family. The family members whose DNA was studied are indicated in grey

To study the paternal lineage DNA profiles of the putative remains of Emperor Nicholas II and Prince Alexey, the STR-haplotypes of the Y-chromosome were determined. Specialized procedures were developed in order to increase the PCR sensitivity, since the amount of the available DNA was limited, and the molecules were highly fragmented (some of the methods are described in Table 3) [45]. The STR-profiles were determined from multiple independent PCR amplifications using no less than three different DNA extracts. Only the alleles that were identified in no less than 2 amplifications were considered authentic. A full Y-STR profile for the bone specimen of Skeleton №4 and for the museum samples of Nicholas II's blood was obtained using these criteria. Low-copy highly fragmented DNA often loses single STR alleles. Marker DYS385 shows two loci on the Y-chromosome. The high molecular weight allele (DYS385/ 14) was identified only once in the repeated experiments with the DNA extracted from Sample #146, thus this allele for Sample #146 is indicated as not determined (ND). DNA isolated from the archival Nicholas II bloodstain and DNA obtained from Romanov paternal lineage family members were used as reference samples (Fig. 6). Y-chromosome STR-profiles of the studied samples and the reference sequences were completely identical (Fig. 7 and Table 5). This 17-locus Y-STR haplotype is unique. It is not found in large population databases for multi-locus Y-STR (Table 4) and was first encountered in the described study [45].

**Table 5:** STR-haplotype analysis of the Y-chromosome [45]

Markers	№ 4	№ 146	Members of the Romanov family	Archive blood stain from a shirt	Control DNA ABI, 007
DYS456	16	16	16	16	15
DYS389I	13	13	13	13	13
DYS390	24	24	24	24	24
DYS389II	29	29	29	29	29
DYS458	17	17	17	17	17
DYS19	14	14	14	14	15
DYS385	11, 14	11, ND	11, 14	11, 14	11, 14
DYS393	13	13	13	13	13
DYS391	10	10	10	10	11
DYS439	11	11	11	11	12
DYS635	24	24	24	24	24
DYS392	13	13	13	13	13
Y-GATA-H4	12	12	12	12	13
DYS437	15	15	15	15	15
DYS438	12	12	12	12	12
DYS448	19	19	19	19	19

**Fig. 8.**
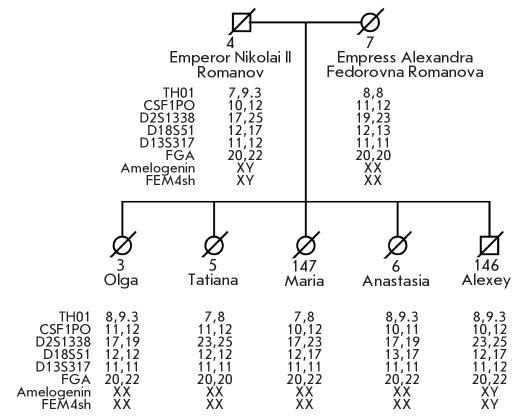
Analysis of the sex chromosome and autosomal STR-markers in DNA extracted from bone specimens [45]

Further gender and autosomal chromosome genotyping with STR multiplex systems developed especially for degraded DNA demonstrated that the male (Sample № 146) and the female (Sample № 147) from the second grave have autosomal STR profiles nonidentical to any STR profiles determined for Romanov family remains from the first grave but consistent with a biological kinship connection ((Fig. 8). These data clearly demonstrate that these newly found remains may belong to Prince Alexey and one of the daughters of the imperial family. The available nuclear DNA analysis data, supported by the anthropological data, prove that the remains from the second grave belong to a young woman (№ 147) and a boy (№ 146), and that samples from the second grave are not from Skeleton № 7 (putative mother, Empress Alexandra Fedorovna) or Skeleton № 4 (putative father, Emperor Nicholas II); however, they are related through the paternal and maternal lineages.

The statistical evaluation (likelihood ratio) based on three identification approaches for determining whether the bones belong to Nicholas II, and not to any other random individual, is on the order of a septillions (> 10^26^) [45]. Taken together our data establish beyond reasonable doubt that the studied remains belong to the last Russian Emperor Nicholas II Romanov, his wife the Empress Alexandra Fedorovna, their 4 daughters (the Grand Duchesses Olga, Tatiana, Maria, and Anastasia), and their son (Prince Alexey).

## HEMOPHILIA: SEARCH FOR MUTATIONS IN THE GENES FOR BLOOD CLOTTING FACTORS

There is historical evidence that Prince Alexey suffered from severe bleeding that is characteristic of hemophilia. It is now known that hemophilia is caused by insufficient activity of blood clotting factors. Factor VIII deficit caused by mutations in the F8 gene is the cause of the most common hemophilia A (about one in 5 000 boys is born with this disease), and Factor IX deficit causes hemophilia B (F9 gene), which occurs 5 times less often. 

A few hundred mutations that cause hemophilia have been described to this day. Both of the blood clotting factor genes are localized on the X-chromosome; that is why males carrying the mutant gene exhibit the disease. Females carrying a single copy of the mutant gene and a copy of the normal one are healthy in most cases, although some of them (10 %) can exhibit decreased efficiency of blood clotting. Females can be assessed for hemophilia mutations when their sons have hemophilia.

**Fig. 9.**
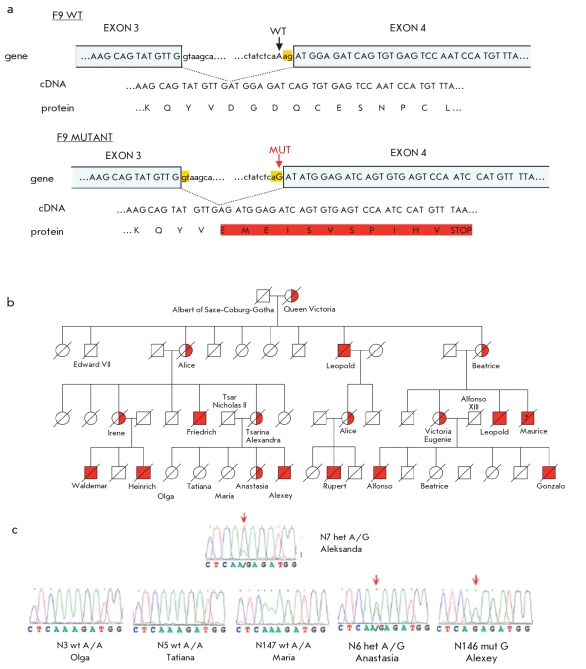
Hemophilia in the royal families of Europe: a - the point mutation in the F9 gene of the bloodclotting factor, that creates an alternative splicing site [49]; b - the pedigree demonstrating the inheritance of hemophilia from Queen Victoria to her descendants; c - sequencing chromatograms of the F9 gene sequence obtained during the analysis of DNA extracted from bone remains

This inherited disease was common in the royal families of Europe, the sons, grandsons, and great grandsons of Queen Victoria (Fig. 9, c). The Queen herself did not suffer from this illness, but it seems that she carried the mutant gene. There is no evidence of hemophilia in any of her present living relatives.

In order to determine whether Alexandra Fedorovna or Prince Alexey carried any mutations in genes, all the exons and the intron-exon boundaries of F8 or F9 genes were amplified using multiplex amplification reaction and then sequenced by massively parallel sequencing. Miniscule amounts of DNA and its high level of degradation required special procedures for the identification of the nucleotide sequences that included the 26 exons of the F8 gene and the 8 exons of the F9 gene (the strategy and methods used in this study are described in Table 6).

**Table 6:** Methods used in screening mutations of the F8 and F9 genes [49]

Stage of analysis	Special procedures	Reagents and methods
Multiplex PCR-amplification of the Factor VIII gene, F8 (26 exons) and Factor IX, F9 (8 exons), both of which are located on the X-chromosome	Extracts which were shown to be free of contamination by foreign samples based on the analyses of mtDNA and STR-markers were used for analyzing the nuclear genes. Just as in the previous analyses, negative controls were used: PCR-amplification of "empty" extracts (obtained by performing all of the extraction procedures, but without adding the bone sample) and PCR amplification without the addition of any DNA.	~ 210 pairs of primers were designed for multiplex PCR amplification of short overlapping sequences (< 200 bp), that would cover all the exons and the intron-exons boundaries of the F8 and F9 genes. The primers were grouped into 14 sets for the F8 gene and 3 sets for the F9 gene, with each set consisting of 7 to 30 pairs of primers. About 100 pg of human DNA (~ 16-17 diploid genomes) were used for the initial multiplex PCR.
Sequencing	Sequencing of the blood clotting factor genes F8 and F9 was done in parallel with the mitochondrial genome to have a control for contamination and unequivocal identification of the sample.	Individual PCR-fragments were excised and purified from a 2.5 % agarose gel and then sequenced using two strategies. One involved mixing the PCR products in equimolar amounts and using them for sequencing (Illumina GA). The other approach involved the direct sequencing of individual PCR-products on a 96-capillary sequencer 3730xl DNA analyzer (Applied Biosystems).
Genotyping of the identified F9 gene mutation	8 independent amplifications of the N7 sample (Empress Alexandra) were performed. For other bone samples, 2 to 7 independent extracts were analyzed from each sample.	The mutation which was initially found during the DNA analysis of skeleton N7 was verified by sequencing ultra-short amplicons (63 b.p. and 83 b.p.), which were obtained with specially developed primers. The same primers were used to amplify the DNA from bone fragments N146 (Prince Alexei) and N3, N5, N6, N147 (Nicolas the Second's and Alexandra's daughters).
Analysis of splicing products		The amplified fragment which bore the mutated region of the F9 gene was cloned into the pET01 Exontrap vector (MoBiTec). After verifying the structure of the recombinant molecule by sequencing, it was used to transfect a cell culture. cDNA was obtained from the spliced DNA by using RT-PCR. The cDNA was then amplified, and the PCR product library was sequenced (Illumina GA).The spliced sequences were identified in 812,114 reads. 99.982 % of the transcripts were spliced at the mutant site, and only 0.018 % were spliced at the wild-type site.

The first step was to analyze DNA extracts from Alexandra that showed no contamination based on the results of mtDNA and autosomal STR-marker analysis. Amplification of the F8 gene and the 8 exons of the F9 gene was performed in parallel with the amplification of mtDNA that was used as a control and for accurate identification of the sample. Nonsynonymous substitutions or insertion-deletion mutations were not found in either the F8 or F9 gene. However, we detected a single nucleotide substitution of A =>G at an intron-exon boundary and, 3 nucleotides before the start of the 4^th^ exon of the F9 gene (referred to as IVS3-3A>G according to standard nomenclature). As expected for a heterozygote carrier, Alexandra Fedorovna carried both mutant and wild-type sequences. Alexey bear only the mutant allele, meaning that he was homozygous for this mutation, while one of his sisters (presumably Anastasia) was a heterozygous carrier of the mutation. The other sisters did not carry the mutant alleles; they were homozygous carriers of the wild-type allele (Fig. 9) [49].

Bioinformatic analysis showed that the IVS3-3A>G mutation activates the cryptic splicing acceptor site, which leads to frame-shift during the translation of the F9 gene mRNA and the appearance of a premature stop-codon (Fig. 9, a).

The effect of this mutation on RNA splicing was studied by expressing the mutant fragment of the F9 gene in a cell culture using a specialized recombinant Exontrap vector (MoBiTec). We found that 99.98 % of transcripts were generated by splicing at the mutant site. Less than 1% of the activity of Factors VIII or IX leads to severe manifestations of hemophilia [50]. Population database searches and genotyping of a large cohort of unaffected individuals did not reveal any IVS3-3A>G affected individuals, while we found three reported hemophilic patients with the same substitution in Hemophilia B databases (Table 4). All three had reduced activity of Factor IX (≤1 % of normal activity) and manifested severe hemophilia symptoms. These data confirmed that the hereditary illness in Queen Victoria's lineage, including Prince Alexey, was a severe form of hemophilia B caused by a rare mutation in the F9 gene [49]. Since none of Queen Victoria's ancestors were known to have hemophilia, it can be assumed that this mutation was acquired de novo during gametogenesis in one of her parents.

## CONCLUSION

The methods of DNA analysis developed and widely used in recent years allow experimental identification and reconstruction of DNA nucleotide sequences extracted from biological samples preserved for prolonged periods of time in natural conditions or have been subjected to DNA and its con-stituent molecules damage. The possibility of a successful study of such samples is provided by the use of novel sequencing strategies, novel methods for extracting and purifying DNA, specially equipped facilities, variety of control experiments, and additional methods of data analysis that prevent interpretation errors in the data. All this factors have contributed to the extraction of genetic information from organisms that have disappeared tens of thousands of years ago, and to the reconstruction of evolutionary events, which was hitherto unachievable in experimental study. These findings opened new possibilities for precise molecular genetic analysis of severely damaged and decayed DNA, which has already raised the standard of ap-plied procedures in forensic medicine. The results reviewed in this paper could not have been obtained without the development of novel DNA tech-nologies that can now be incorporated into the everyday routines of fundamental and applied research, making them more reliable, fast and informa-tive, as well as lowering costs.

## Acknowledgements

The study was supported by the Russian Federal Agency for Science and Innovation (federal contract 02.512.11.2231) and by the Program "Biodiversity" of Presidium of Russian Academy of Sciences.
